# Population-based incidence of all-cause anaphylaxis and its development over time: a systematic review and meta-analysis

**DOI:** 10.3389/falgy.2023.1249280

**Published:** 2023-12-12

**Authors:** Vanessa Pühringer, Bernd Jilma, Harald Herkner

**Affiliations:** ^1^ Department of Clinical Pharmacology, Medical University of Vienna, Vienna, Austria; ^2^ Department of Emergency Medicine, Medical University of Vienna, Vienna, Austria

**Keywords:** anaphylaxis, epidemiology, incidence, meta-analysis, systematic review

## Abstract

**Introduction:**

It is extremely difficult to compare studies investigating the frequency of anaphylaxis making it challenging to satisfactorily assess the worldwide incidence rate. Using a systematic review and meta-analysis, this publication aims to determine the current incidence of all-cause anaphylaxis worldwide. Additionally, we investigated whether the incidence of anaphylaxis has changed over time and which factors influence the rates determined by individual studies.

**Methods:**

A literature search was performed in four databases. All articles that reported relevant information on population-based incidence rates of all-cause anaphylaxis were included. The protocol was published on INPLASY, the International Platform of Registered Systematic Review and Meta-analysis Protocols.

**Results:**

The database query and screening process resulted in 46 eligible articles on anaphylaxis. The current incidence worldwide was found to be approximately 46 cases per 100,000 population per year (95% CI 21–103). Evaluating confounding factors showed that studies using allergy clinics and hospitalizations as data source result in comparably low rates. Moreover, children are less prone to develop anaphylaxis compared to the general population. Using a random effects Poisson model we calculated a yearly increase of anaphylaxis incidence by 7.4% (95% CI 7.3–7.6, *p* < 0.05).

**Discussion:**

This seems to be the first approach to analyze every reported all-cause anaphylaxis incidence rate until 2017 for an at most accurate determination of its epidemiology. Based on these results, future research could investigate the underlying causes for the rising incidence in order find ways to decrease the condition’s frequency.

**Systematic Review Registration:**

inplasy.com, identifier [INPLASY202330047]

## 1. Introduction

Anaphylaxis, a condition which was first described approximately 100 years ago (1), can be summarized as an acute, systemic and very severe type of allergic reaction. Similarly to other allergic reactions, anaphylaxis most frequently works through an IgE (immunoglobulin E) -mediated immunologic mechanism. After binding of the allergen to the allergen-specific IgE receptor (Fc*ε*RI) complex, IgE triggers the release of inflammatory mediators (e.g., histamine, tryptase) and cytokines from mast cells and basophils. This leads to immunologic reactions such as vasodilation and increased vascular permeability (2–4). Non-IgE mediated anaphylaxis can be immunologic or non-immunologic (earlier referred to as anaphylactoid reactions) and present the same way as IgE-mediated anaphylaxis. According to the criteria defined by the NIAID/FAAN (National Institute of Allergy and Infectious Disease and the Food Allergy and Anaphylaxis Network) Symposium, the clinical picture of anaphylaxis varies significantly. It may involve a combination of cutaneous, respiratory, cardiovascular and gastrointestinal symptoms, as well as organ dysfunction and hypotension (2, 3, 5).

There is no universally accepted definition of anaphylaxis (2, 6–9). This makes it difficult to compare the results of studies and determine the worldwide population-based incidence rate (10–12). Other factors could also influence the calculated incidence rate (13–15). These include the study design applied [e.g., prospective or retrospective, patient hospitalization vs. ED (emergency department) attendance] and the analyzed area. Some researchers retrieve cases with anaphylaxis related ICD (International Classification of Diseases) codes from national databases without further review of patient records (16–18). Others apply published diagnostic criteria for anaphylaxis (19–21), e.g., the definition established by the NIAID/FAAN Symposium (2).

A considerable number of publications claim that the incidence of anaphylaxis is increasing over time (10, 11, 22–25). Other articles, however, state that the incidence relative to the included number of inhabitants is constant (26) or even decreasing (27). Turner et al. (28) found a steady increase in hospital admissions due to anaphylaxis from 1992 to 2008 but a rather constant incidence rate from 2008 to 2012. Anderson et al. (29) reported an increasing anaphylaxis incidence in patients aged below 20 but a constant incidence rate for all patients aged 20 years and older.

Knowing the incidence of anaphylaxis allows a better analysis of trends over time. This can be used to monitor the impact of changes introduced in anaphylaxis guidelines, of new diagnostic possibilities or hospital admission regulations (30). Anaphylaxis can be caused by various allergens, including food products, drugs, insect venoms, exercise or latex. Sometimes these reactions are caused by multiple factors, or the responsible proteins remain unidentified (31, 32). An overview on the triggers and related mechanisms of anaphylaxis is given in the World Allergy Organization (WAO) Guidance 2020 (5). Anaphylaxis is also frequently found in patients with hereditary alpha tryptasemia and/or clonal mast cell disorders (33, 34), which can lead to particularly severe, recurrent or protracted anaphylaxis (35, 36).

This article investigates the incidence of all-cause anaphylaxis. With the help of the PRISMA (Preferred Reporting Items for Systematic reviews and Meta-Analyses) guideline (37), this systematic review and meta-analysis aims to determine the current population-based incidence rates of anaphylaxis worldwide and per continent. Additionally, it evaluates whether the incidence has increased during the past decades. By analyzing confounding factors, potential underlying reasons for the wide range of reported incidence rates are sought to be identified.

## 2. Methods

The protocol for this systematic review and meta-analysis was registered and published on the International Platform of Registered Systematic Review and Meta-analysis Protocols (https://inplasy.com/) with the registration number INPLASY202330047. The manuscript was prepared following the PRISMA Statement (37).

### 2.1. Data sources

A systematic query combining anaphylaxis and epidemiology related terms was performed with four electronic databases, i.e., MEDLINE (RRID:SCR_002185, by OVID), EBSCO CINAHL [(Cumulative Index to Nursing and Allied Health Literature), RRID:SCR_022707, by EBSCOhost], Web of Science (RRID:SCR_022706) Core Collection (by Clarivate Analytics) and LILACS (Latin American and Caribbean Health Science Information).

### 2.2. Search strategy

The search strategies applied in each of these databases are shown in the Supplementary Material of this article (Supplementary Table S1–S4). The search strategy was designed for MEDLINE based on a publication by Panesar et al. (38). It was then adapted for use in the three other databases. As the aim was to assess the development of incidence over time, no limitation on the year of publication was set.

### 2.3. Screening methodology

**Figure 1:**
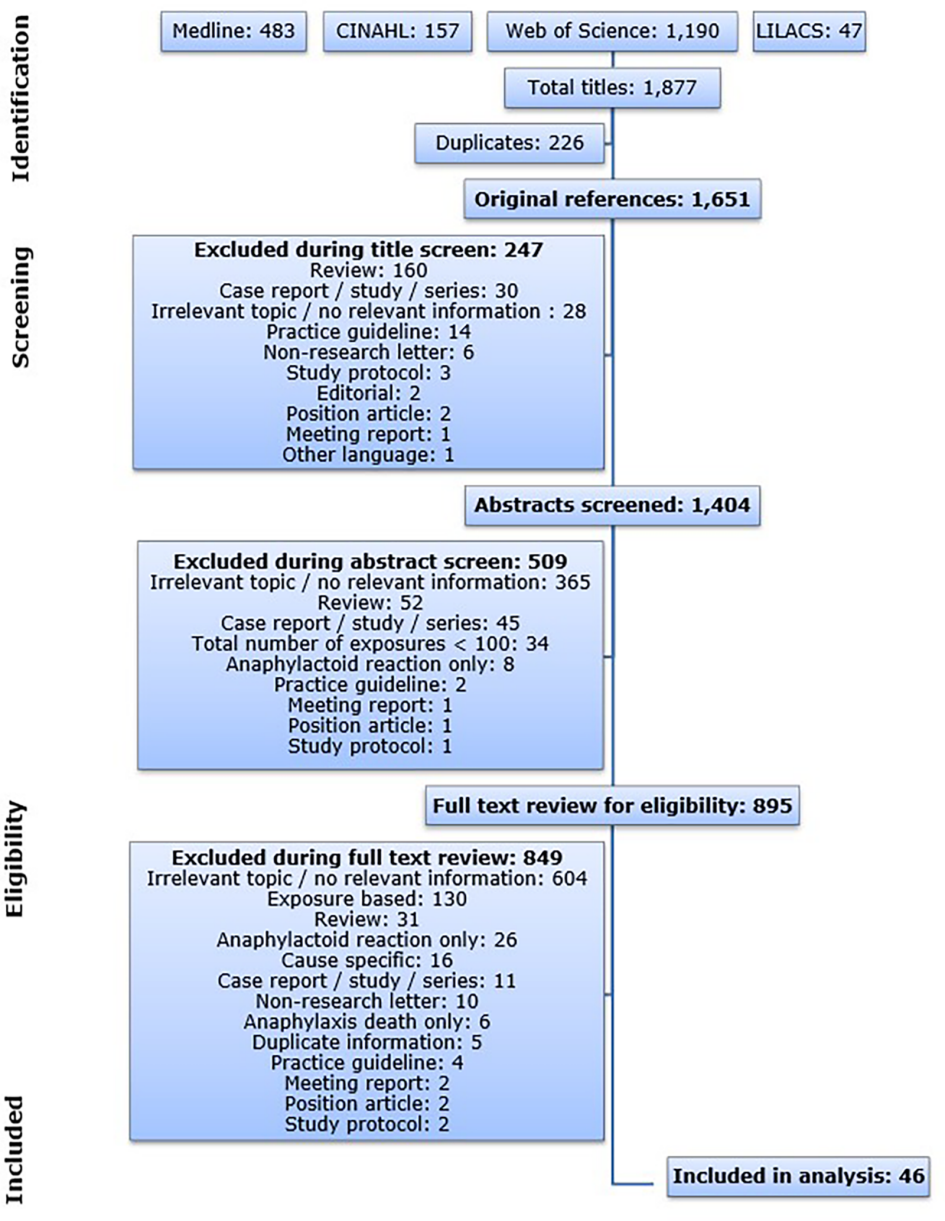
Flow diagram illustrating the database search process and the subsequent article screen. CINAHL: Cumulative Index to Nursing and Allied Health Literature; LILACS: Latin American and Caribbean Health Science Information Database.

The publications resulting from the database queries were imported into the Reference Manager program (version 11). Automatic and manual duplicate checks were performed to eliminate publications retrieved from multiple databases. The resulting publications were screened based on the criteria below. The flow diagram illustrating the search and screening process is shown in Figure 1.

Some publications were already excluded based on the title or on the publication type indicated on the NCBI (National Center for Biotechnology Information, RRID:SCR_006472) website (https://www.ncbi.nlm.nih.gov). Other publications were excluded after reading the abstract and for others reading the full text was essential. Due to the large amount of articles that were retrieved from the databases, the screening was performed by one person and a subsequent quality check by spot samples. For all instances during the screening procedure: In case a specific level of screening did not give enough information to allow evaluation of eligibility, the publication was taken to the next level in order to prevent risk of selection bias. All articles that did not meet any of the exclusion criteria and that included relevant information on population-based incidence rates of all-cause anaphylaxis were included in this analysis.

### 2.4. Eligibility criteria and study selection

In general, the following study designs were included: cohort studies, cross-sectional studies, case control studies, randomized controlled trials and pharmacovigilance studies. Reviews, discussions, non-research letters, editorials, practice guidelines, study protocols, position articles, meeting reports, case reports, case studies, case series and animal studies were, on the other hand, excluded. This concept is similar to the one published by Panesar et al. (38). Whilst some of these study designs were already included or excluded via the search parameters in the mentioned databases, an additional selection process was necessary during the article screen.

For this analysis, only all-cause population-based incidence rates of anaphylaxis were included—i.e., publications where the authors stated the reactions were anaphylactic. Hence, publications which reported on non-IgE-mediated reactions or ‘anaphylactoid reactions’ were excluded. The same is true for results on prevalence or exposure based frequencies, as well as for publications reporting on cause specific incidence rates or solely on anaphylaxis caused death.

In cases where research was performed on a population of a specific age range, the publication was included if the size of the respective reference population was available or if the incidence rate was readily provided in the article. These publications are indicated via footnotes under Supplementary Table S6. Articles for which the size of the reference population was not known could not be included. As far as language is concerned, articles written in English or German were included.

### 2.5. Risk of bias assessment

For all publications included in the analysis, a risk of bias assessment was undertaken by using a set of ten questions. Those involved both internal and external validity plus a summary rating (39). The analysis was performed by two independent reviewers. Disagreement was resolved by discussion.

### 2.6. Data extraction

The following variables were extracted for the incorporated publications: period of analysis, country of analysis, size of reference population, data source, anaphylaxis definition, incidence per 100,000 population per year and, if applicable, population age range. These data are outlined in Supplementary Table S6, S7. In many articles the incidence rate of anaphylaxis *per se* was not stated. In this case the rate was calculated using the data available. If necessary, population sizes of each year studied were looked up on given websites, and the resulting average number of inhabitants was used for the incidence rate calculation.

### 2.7. Meta-analysis

This study calculated the current population-based incidence rate of all-cause anaphylaxis worldwide, analyzed its development over time, and compared the incidence rates between the investigated continents. Moreover, key characteristics of included articles (i.e., data source or population age range) were investigated as confounding factors. Poisson regression was used to model incidence rates. To allow for within and between study variability we used random effects models. Average event rates across all studies were calculated using a plain random effects Poisson model. Potential predictors of event rates were used as covariates in these models. For predictors on a continuous scale we modelled these on the original scale as well as indicator variables. The likelihood ratio test was used to test deviations from linearity. A bubble plot of the association between years and event-rate was generated, weighted for the inverse variance of the individual studies’ estimates. If the rate published in an article was not specific to one year but concerned a period, the middle year of this period was used for the chart. For data management and analyses Microsoft Excel (RRID:SCR_016137) and Stata (RRID:SCR_012763) 14.2 were used. Generally, a two-sided *p*-value <0.05 was considered statistically significant.

## 3. Results

### 3.1. Database query and article screen

The database queries resulted in a total of 1,877 publications and 1,651 publications after all duplicates were removed. The article screen identified 46 articles that met the predefined inclusion criteria published between 1993 and 2017 (Figure 1). The 46 publications on anaphylaxis contained 119 individual incidence rates. All eligible articles were published in English.

### 3.2. Risk of bias

The risk of bias assessments done according to Hoy et al. (39) resulted in 59% (*n* = 27) of studies with low, 35% (*n* = 16) with moderate and 7% (*n* = 3) with high risk of bias. In the majority of articles, the representativeness of the national population and the target population as well as an insufficiently proven reliability of the study instrument have led to an increased risk of bias. Details are shown in Supplementary Table S5.

### 3.3. Sources of data and anaphylaxis definition

A high number and wide range of incidence rates (from 0.49 to 328.7 cases per 100,000 population per year) for all-cause anaphylaxis was received. The studies used hospitalizations or admissions, emergency departments (EDs), multiple care providers and epinephrine prescriptions as source for data. All data points related to “allergy clinics, specialists and EDs” (40) and to a health maintenance organization database (18) were reported from the same publications, respectively.

Additionally to the data sources, the applied definitions of anaphylaxis were investigated and presented in Supplementary Table S7. In summary, several studies searched based on ICD codes (*n* = 33) or text strings (*n* = 7) entered in healthcare databases. Thereby, 22 restricted their query to terms specific for anaphylaxis and 18 included also signs and symptoms. Four studies used a code-based algorithm by Harduar–Morano et al. (41). In articles where patient records were manually reviewed, nine were based on self-defined criteria and nine on published recommendations for anaphylaxis definitions, such as the NIAID/FAAN anaphylaxis criteria (3) or the WAO anaphylaxis guidelines (42). The authors of three publications measured the incidence of anaphylaxis based on the number of epinephrine prescriptions. Even though total mast cell tryptase is a serologic marker that can assist in the diagnosis of adult anaphylaxis (43), tryptase levels were not part of the anaphylaxis definitions for the data used in our analysis. Overall, 37 different definitions of anaphylaxis were used out of the 46 publications incorporated in our meta-analysis.

### 3.4. Current anaphylaxis incidence rate

An attempt was made to identify the worldwide incidence of all-cause anaphylaxis. The weighted approximate rate was found to be 44 cases per 100,000 population per year (σ = 136). After that, the articles were grouped into time quintiles based on their years of analysis in a way that all quintiles contained an equal amount of data points. It is more applicable to consider only the past quintile (years of analysis 2010–2014) for the calculation of a currently applicable incidence rate, which was determined to be 46 cases per 100,000 population per year (95% CI 21–103).

As an additional step, the incidence rates were calculated specifically for each continent of analysis based on all eligible articles: The results per 100,000 population per year were as follows: 8 cases for Asia, 17 cases for Australia, 43 cases for North America and 71 cases for Europe. Supplementary Figure 2 presents a forest plot related to these four continents. The average incidence rate for South America could not be calculated due to the small amount of data available: Hoyos–Bachiloglu et al. (44) reported 1.41 cases per 100,000 population per year in Chile. In one publication (45) the country or continent of analysis was not mentioned but the authors are affiliated with the Mayo Clinic in the United States (9.69 cases per 100,000 population per year).

Within each continent a north-south gradient of incidence rates could not be identified (Supplementary Table S6). Indeed countries had similarly high and low incidence rates, regardless of their location in the north or the south.

Another statistical analysis revealed that when adjusting for the changes in years, the effect on the continent differences is not changed. The same is true for the effect of the difference in continents on the change in years.

### 3.5. Development of anaphylaxis incidence over time

**Figure 2:**
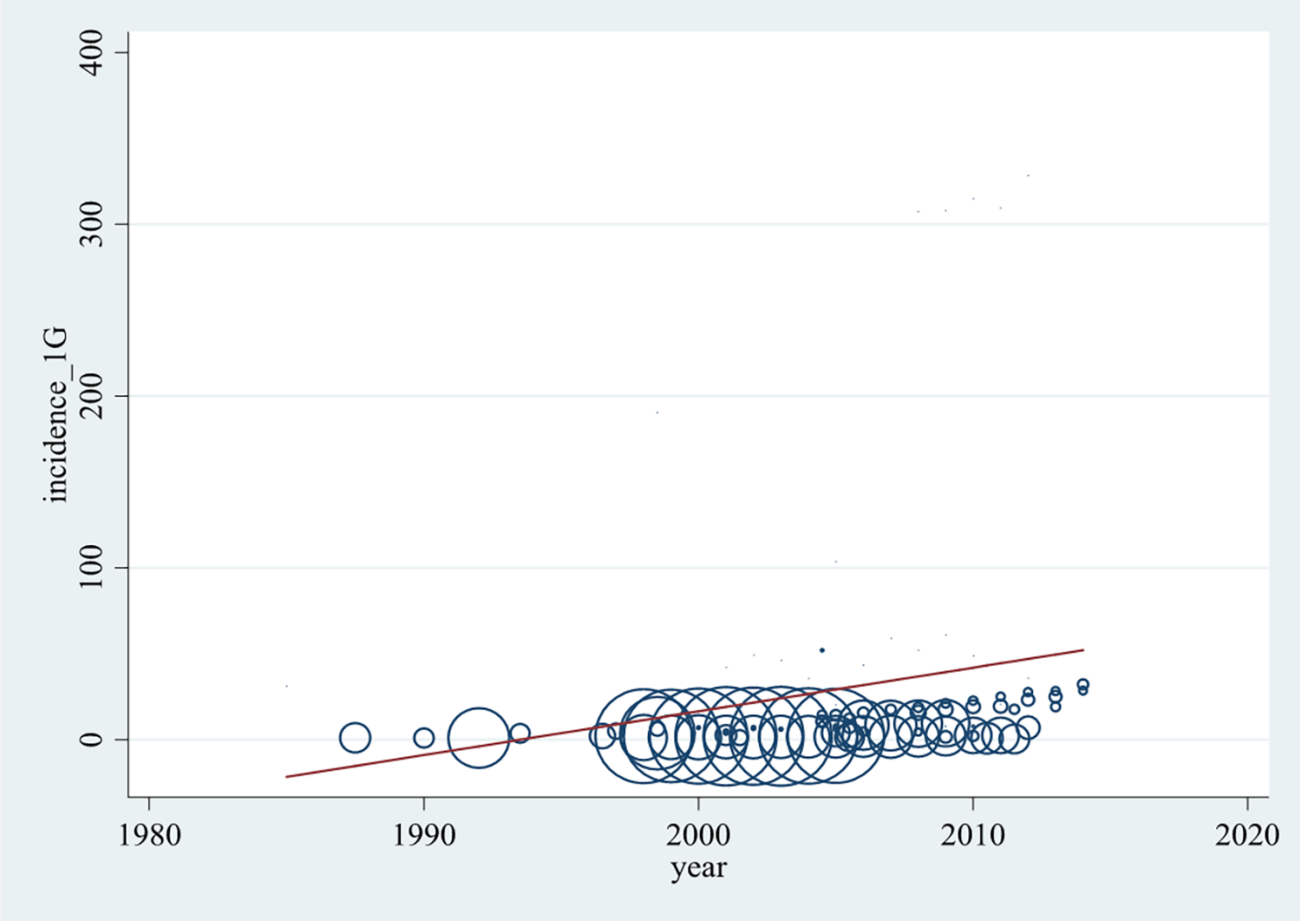
Development of all-cause anaphylaxis incidence (per 100,000 population per year) over time. Each data point represents an annual rate reported by the original articles. The larger bubble, the higher weighted the data point is.

In order to determine whether the anaphylaxis rate has increased in the past decades, a bubble plot was created (Figure 2). It presents the incidence rates of all incorporated articles per 100,000 population per year against the years of analysis (1983–2014) including a trend line.

As seen in Figure 2 and Supplementary Table S6, incidence rates were available for years of analysis between 1983 and 2014. The highest density of data is available between 2000 and 2010. Most reported incidence rates of anaphylaxis lie between 0 and 30, while several lie between 30 and 62 anaphylaxis cases per 100,000 population per year. From the publications by Simons et al. (46) and Tejedor Alonso et al. (20) rates between 100 and 200, and from Pourang et al. (18) rates higher than 300 cases per 100,000 population per year were determined. The higher weighted publications were found to report comparably low incidence levels, thus the rates of >100 cases per 100,000 population per year are illustrated by relatively small bubbles.

Although the association represented by the trend line is not satisfactorily described with a linear model, Figure 2 shows a significant tendency of anaphylaxis increase over the past decades.

The subsequently performed random effects Poisson regression demonstrated a yearly rise of all-cause anaphylaxis incidence by 7.4% (95% CI 7.3–7.6, *p* < 0.05) and a 25.0% (95% CI 24.4–25.6, *p* < 0.05) rate increase per time quintile. This result is not influenced by potential outlier values, which was evaluated by a sensitivity analysis that excluded all incidence values >300 cases per 100,000 population obtained for the years 2008–2010.

### 3.6. Evaluation of confounding factors

It was evaluated whether any other factors related to the design of the analyzed studies could have an impact on the resulting incidence rate. First, the source of data was analyzed by applying publications that involve multiple clinical settings as basis for calculation: Compared to that, allergy clinics (IRR = 0.1, *p* = 0.02) as well as a composite of admissions, hospitalizations and inpatients (IRR = 0.3, *p* = 0.04) showed significantly lower incidence rates. On the contrary, publications using epinephrine prescriptions (IRR = 1.9, *p* = 0.43), specialist practices (IRR = 8.6, *p* = 0.17) or a health maintenance organization (IRR = 8.9, *p* = 0.07) as data source resulted in almost 2–9 times higher incidence rates. For emergency related sources (e.g., departments, physicians, transports) a similar incidence rate as in publications with multiple clinical settings was found (IRR = 0.9, *p* = 0.9).

Compared to publications that included an entire population (53 cases per 100,000 population per year), the articles that analyzed only pediatric inhabitants (5 cases per 100,000 pediatric population per year, IRR = 0.1, *p* < 0.05) or only adult inhabitants (20 cases per 100,000 adult population per year, IRR = 0.4, *p* = 0.24) resulted in lower incidence rates. Consequently, children are significantly less prone to suffer from anaphylaxis than the general population. A forest plot is given in Supplementary Figure S2.

## 4. Discussion

The main aim of this study, which was compiled taking into account the PRISMA guideline (37), was to identify the current population-based incidence rate of all-cause anaphylaxis worldwide using a systematic review and meta-analysis. The publications analyzed were very heterogeneous by rarely using the same study design or definition of anaphylaxis, which complicated the comparability of the study results. In the 46 articles included in this meta-analysis, 37 different definitions of anaphylaxis were used. This high variability is a known challenge in anaphylaxis research (10–12).

The results of our incidence analysis ranged from 0.49 to 328.7 anaphylaxis cases per 100,000 population per year. This wide range of incidence rates, even when using a similar analysis period, was also found in the systematic review by Panesar et al. (30). Three publications (18, 20, 46) reported incomparably high incidence rates (i.e., >100 cases per 100,000 population per year); however, these studies applied differing methods of analysis. Simons et al. (46) determined the incidence of anaphylaxis in Canada based on the number of epinephrine prescriptions. Their study showed a high incidence rate, although only one epinephrine dispensing per individual was applied for the calculation. Nevertheless, physicians could have prescribed epinephrine for sensitization therapy, as a precaution for positive skin test or *in vitro* test results or for symptoms similar to anaphylaxis (46). Pourang et al. (18), on the other hand, investigated the incidence of anaphylaxis in California by searching a health maintenance organization database for anaphylaxis related ICD codes.

Tejedor Alonso et al. (20) used a database that incorporated data from various clinical settings (EDs, hospitalizations, allergy clinics, etc.) in Spain. After a search for anaphylaxis related keywords, they additionally reviewed the patient records according to the NIAID/FAAN criteria of anaphylaxis (2). In another study (47), the authors investigated only the admissions and retrieved anaphylaxis cases using a validated ICD-9-CM (Clinical Modification) code algorithm (41) without performing further review of patient charts. The resulting incidence rates were substantially lower (i.e., approximately 1.8 cases per 100,000 population per year) than in their previous publication in 2012 (20). In a third study by Tejedor Alonso et al. (48), the incidence rate was as low as 1.7 cases per 100,000 population per year. In this study an admissions database was searched for both specific and unspecific anaphylaxis codes. After this a manual review was performed. By looking at the methodology of these three studies, one could argue that the discrepancy in incidence rates results from using key words instead of ICD codes. This is, however, rather unlikely because the medical review of patient charts would have eradicated incorrectly classified cases. Instead, the high incidence rate in their first study (48) presumably comes from the fact that multiple clinical settings, rather than only admissions, were included. Other studies discussed in this systematic review (49–51) used databases incorporating all care providers and obtained incidence rates between 15.6 and 61.4 cases per 100,000 population per year.

The above-described findings are supported by the statistical examination of confounding factors within this meta-analysis: it does not come surprising that the incidence rates solely based on data from allergy clinics or hospitalizations were significantly lower than those involving multiple clinical settings. Importantly, the meta-analysis revealed higher incidence rates for publications that used epinephrine prescriptions, specialist practices or a health maintenance organization only as their data source.

The current incidence of anaphylaxis was determined with meta-analysis by taking into account rates assigned to the last 5 years of analysis (i.e., 2010–2014), and it resulted in 46 cases per 100,000 population per year. The rather broad associated 95% CI range (i.e., 21–103) is explained by the above mentioned heterogeneity in original study designs and reported individual incidence rates. Similarly as detected by another systematic review focusing on fatal anaphylaxis (52), our study showed a lower incidence rate in children compared to the general population. Dividing the results into the continents that were investigated, the average rates (per 100,000 population per year) were substantially higher in Europe (71 cases) and North America (43 cases) than in Asia (8 cases) and Australia (17 cases). To determine whether the risk of anaphylaxis is truly higher in Europe and North America than in other regions or whether this finding is a result of different thresholds for disease definition, further international cross-continental research is necessary using a harmonized method of analysis.

As authors often claim that the incidence of anaphylaxis might be increasing with time (10, 11, 16–19), this study was designed to investigate the development of anaphylaxis incidence during the past decades. Looking at publications individually, Hananashvili et al. 2016 (21) reported a sudden increase in incidence rates between 2010 and 2011 (from 7.83 to 21.15 cases per 100,000 population per year), which reportedly comes from an increased rate in the Jewish subpopulation studied. A large rise in incidence rates within one year of analysis, typically does not result from an increased number of reactions; it may rather be a consequence of an altered threshold of hospital admission, a changed method of case processing due to the introduction of new diagnostic materials or a new guideline on anaphylaxis definition.

As shown by the bubble plot including weighted rates of individual articles and a connecting trend line, the incidence rate of anaphylaxis has steadily risen by approximately 7.4% per year. Several causes could lead to this observation. An augmented use of allergenic agents, such as peanuts or latex and westernization (53) could result in a true change in the epidemiology of anaphylaxis. Another reason would be an improved recognition and recording system in healthcare (54). According to several national health surveys (55), the number of food allergies in the U.S. has tripled between 1993 and 2006. It was discussed that physicians working in various areas of expertise apply different definitions for food allergies. Thus, overestimation might result from the inclusion of other food-related diseases (e.g., intolerance) or unspecific diagnostic tests (56). Others believe that the rate increase over a relatively short time period was too rapid to be caused by changes in recognition or diagnostic labelling (57), but results from multiple causes.

In general, all study designs that may be used to determine the incidence of anaphylaxis have both advantages and drawbacks, which could lead to over- or underestimation: database queries that include symptom related terms without further review of patient records, or inclusion of non-IgE-mediated reactions due to missing immunologic tests, will lead to inclusion of false-positive cases and overestimation. In contrast, searching only specific anaphylaxis terms might lead to underestimation of anaphylaxis frequency due to the known under-diagnosis (e.g., mild anaphylaxis, anaphylaxis death) and under-reporting of this acute reaction (58–62). A further potential for underestimation is the use of databases capturing only ED visits or hospitalizations (27) rather than national healthcare databases, as done in the majority of articles included in this systematic review. For the above-mentioned reasons, the incidence rates of anaphylaxis will often be over- or underestimated. This systematic review presents a meta-analysis of a large number of results since 1983 and therefore attenuates these deviations.

Few systematic reviews assessing the incidence of anaphylaxis in a population-based manner have been published thus far. Panesar et al. (38) limited the area of analysis to Europe and the publication period to 2000–2012 and identified a range of incidence rates between 1.5 and 7.9 per 100,000 person-years. Umasunthar et al. (63) focused exclusively on food induced anaphylaxis. The American College of Allergy, Asthma and Immunology Epidemiology of Anaphylaxis Working Group also investigated the frequency of anaphylaxis. However, the included articles were not based on a systematic database query but were selected by the committee, a method which could increase the risk of selection bias (12). The authors identified the frequency of anaphylaxis to be approximately 50–2,000 episodes per 100,000 persons. Wang et al. (64) investigated the worldwide incidence of anaphylaxis specifically in children and also yielded a wide range of incidence rates (from 1 to 761 per 100,000 person-years). Perez-Codesido et al. (52) published a systematic review after analyzing observational studies specifically on fatal anaphylaxis and determined a mortality rate of 0.002–2.51 deaths per million person-years.

Our study looked at systematically retrieved articles concerning all-cause anaphylaxis without limitation on time of publication, country of analysis or population age group to receive the most complete picture possible. The differences in case definitions and data sources used in the original articles, on the other hand, may have led to an increased confounding of the results. Furthermore, as literature in the past differentiated between anaphylaxis and non-IgE mediated “anaphylactoid reactions”, this study focused on IgE-mediated anaphylaxis. However, other pathways such as the mast cell-specific receptor Mas-related G protein-coupled receptor X2 (MRGPRX2) activation can also trigger events with this clinical picture (65). Another limitation is the fact that the screening of literature articles was performed by only one person and a subsequent quality check by spot samples rather than using a two-eye principle due to the large amount of articles that were retrieved from the databases. This, however, seems to be the first study to determine the development of anaphylaxis incidence over multiple decades by pooling and analyzing the results of all systematically retrieved anaphylaxis incidence reports using meta-analysis.

In conclusion, this systematic review and meta-analysis demonstrated that the frequency of anaphylaxis in the population worldwide has increased over the past decades, and it revealed a current incidence rate of approximately 46 cases per 100,000 population per year. Further research is necessary to uncover the underlying causes for this increased incidence. Knowing the target cause will be the basis for developing strategies to minimize the number of future reactions.

## Data Availability

The original contributions presented in the study are included in the article/**Supplementary Material**, further inquiries can be directed to the corresponding author.
