# Supplementary material for: Population-based incidence of all-cause anaphylaxis and its development over time: a systematic review and meta-analysis
Source: Front Allergy. 2023 Dec 12;4:1249280. doi: 10.3389/falgy.2023.1249280 (PMC10749935; doi:10.3389/falgy.2023.1249280)
Supplement: Supplementary file 1 [file Datasheet1.docx]

Supplementary Material

# Supplementary Tables

## Supplementary Table 1. Search strategy for Medline

| Search # | Search term |
| --- | --- |
| 1 | Anaphylaxis/ |
| 2 | anaphylaxis.mp. |
| 3 | anaphylactic*.mp. |
| 4 | anaphylactoid*.mp. |
| 5 | acute systemic allergic react*.mp. |
| 6 | or/1-5 |
| 7 | (rat? or cow? or bovine or cattle or chicken? or horse? or mice or mouse or murine* or pig? or dog? or animal*).ti. |
| 8 | exp animals/ not humans.sh. |
| 9 | 7 or 8 |
| 10 | 6 not 9 |
| 11 | *Incidence/ |
| 12 | *Prevalence/ |
| 13 | (incidence or prevalence or epidemiol*).ti. |
| 14 | epidemiologic methods/ |
| 15 | *cohort studies/ |
| 16 | *Cross-Sectional Studies/ |
| 17 | controlled clinical trial.pt. |
| 18 | *case-control studies/ |
| 19 | *Randomized Controlled Trials as Topic/ |
| 20 | or/11-19 |
| 21 | 10 and 20 |

## Supplementary Table 2. Search strategy for CINAHL

| Search # | Search term |
| --- | --- |
| 19 | S9 and S18 |
| 18 | S10 or S11 or S12 or S13 or S14 or S15 or S16 or S17 |
| 17 | (MH "Randomized Controlled Trials") |
| 16 | (MH "Case Control Studies") |
| 15 | (MH "cross sectional studies") |
| 14 | (MH "Epidemiology") |
| 13 | (MH "Epidemiological Research") |
| 12 | TI (incidence or prevalence or epidemiol*) |
| 11 | (MH "*Prevalence") |
| 10 | (MH "*Incidence") |
| 9 | S5 not S8 |
| 8 | S6 or S7 |
| 7 | (MH "Animals, Laboratory") |
| 6 | TI (rat? or cow? or bovine or cattle or chicken? or horse? or mice or mouse or murine* or pig? or dog? or animal*) |
| 5 | S1 or S2 or S3 or S4 |
| 4 | AB „anaphylactoid*“ |
| 3 | AB "anaphylactic*" |
| 2 | AB "anaphylaxis" |
| 1 | (MH "Anaphylaxis") |

## Supplementary Table 3. Search strategy for Web of Science™ Core Collection

| Search # | Search term |
| --- | --- |
| 9 | #5 AND #6  Refined by: DOCUMENT TYPES: ( ARTICLE ) AND RESEARCH AREAS: ( ALLERGY OR IMMUNOLOGY OR GENERAL INTERNAL MEDICINE OR PEDIATRICS OR PHARMACOLOGY PHARMACY OR ANESTHESIOLOGY OR PUBLIC ENVIRONMENTAL OCCUPATIONAL HEALTH OR SURGERY OR FOOD SCIENCE TECHNOLOGY OR LIFE SCIENCES BIOMEDICINE OTHER TOPICS OR EMERGENCY MEDICINE OR RESEARCH EXPERIMENTAL MEDICINE OR NUTRITION DIETETICS OR HEMATOLOGY OR CARDIOVASCULAR SYSTEM CARDIOLOGY OR TOXICOLOGY )  Indexes=SCI-EXPANDED, SSCI, A&HCI, CPCI-S, CPCI-SSH, ESCI Timespan=All years |
| 8 | #5 AND #6  Refined by: DOCUMENT TYPES: ( ARTICLE )  Indexes=SCI-EXPANDED, SSCI, A&HCI, CPCI-S, CPCI-SSH, ESCI Timespan=All years |
| 7 | #5 AND #6  Indexes=SCI-EXPANDED, SSCI, A&HCI, CPCI-S, CPCI-SSH, ESCI  Timespan=All Years |
| 6 | TS=(epidemiol* or *incidence or *prevalance or "*cohort stud*" or "*cross$sectional stud*" or "controlled clinical trial$" or "*case$control stud*" or "*Randomi?ed controlled Trial$")  Indexes=SCI-EXPANDED, SSCI, A&HCI, CPCI-S, CPCI-SSH, ESCI  Timespan=All Years |
| 5 | #1 not #4  Indexes=SCI-EXPANDED, SSCI, A&HCI, CPCI-S, CPCI-SSH, ESCI  Timespan=All Years |
| 4 | #2 or #3  Indexes=SCI-EXPANDED, SSCI, A&HCI, CPCI-S, CPCI-SSH, ESCI  Timespan=All Years |
| 3 | TS=(animal*)  Indexes=SCI-EXPANDED, SSCI, A&HCI, CPCI-S, CPCI-SSH, ESCI  Timespan=All Years |
| 2 | TI=(rat? or cow? or bovine or cattle or chicken? or horse? or mice or mouse or murine* or pig? or dog? or animal*)  Indexes=SCI-EXPANDED, SSCI, A&HCI, CPCI-S, CPCI-SSH, ESCI  Timespan=All Years |
| 1 | TS=(anaphylaxis or anaphylactic* or anaphylactoid* or "acute systemic allergic react*")  Indexes=SCI-EXPANDED, SSCI, A&HCI, CPCI-S, CPCI-SSH, ESCI  Timespan=All Years |

## Supplementary Table 4. Search strategy for LILACS

| Search # | Search term |
| --- | --- |
| 1 | TW:(anaphylaxis or anaphylactic or anaphylactoid) AND TW:(incidence OR prevalence OR epidemiol$) AND NOT TI:(allerg$) |

## Supplementary Table 5. Risk of bias assessment

| Study | Q1 | Q2 | Q3 | Q4 | Q5 | Q6 | Q7 | Q8 | Q9 | Q10 | Overall risk of bias |
| --- | --- | --- | --- | --- | --- | --- | --- | --- | --- | --- | --- |
| Anandan et al. 2009 (1) | ✓ | X | ✓ | ✓ | ✓ | ✓ | X | ✓ | ✓ | X | Moderate |
| Beyer et al. 2012 (2) | X | X | ✓ | X | ✓ | ✓ | X | ✓ | ✓ | X | High |
| Brown et al. 2001 (3) | X | X | ✓ | ✓ | ✓ | ✓ | ✓ | ✓ | ✓ | ✓ | Low |
| Buka et al. 2015 (4) | X | ✓ | ✓ | ✓ | ✓ | ✓ | ✓ | ✓ | ✓ | X | Low |
| Calvani et al. 2008 (5) | X | ✓ | ✓ | ✓ | ✓ | ✓ | ✓ | ✓ | ✓ | ✓ | Low |
| Cetinkaya et al. 2013 (6) | X | X | ✓ | ✓ | ✓ | ✓ | X | ✓ | ✓ | X | Moderate |
| Clark et al. 2014 (7) | X | X | ✓ | ✓ | ✓ | ✓ | ✓ | ✓ | ✓ | ✓ | Low |
| Gaeta et al. 2007 (8) | ✓ | X | ✓ | ✓ | ✓ | ✓ | X | ✓ | ✓ | ✓ | Low |
| Hananashvili et al. 2016 (9) | X | ✓ | ✓ | ✓ | ✓ | ✓ | ✓ | ✓ | ✓ | ✓ | Low |
| Harduar-Morano et al. 2011 (10) | X | ✓ | ✓ | ✓ | ✓ | ✓ | ✓ | ✓ | ✓ | X | Low |
| Helbling et al. 2004 (11) | ✓ | ✓ | ✓ | ✓ | ✓ | ✓ | X | ✓ | X | ✓ | Low |
| Hoyos-Bachiloglu et al. 2014 (12) | ✓ | X | ✓ | ✓ | ✓ | ✓ | ✓ | ✓ | ✓ | ✓ | Low |
| Kivistö et al. 2016 (13) | X | X | ✓ | ✓ | ✓ | ✓ | X | ✓ | ✓ | X | Moderate |
| Lauritano et al. 2013 (14) | X | X | ✓ | ✓ | ✓ | ✓ | ✓ | ✓ | ✓ | ✓ | Low |
| Lee et al. 2017 (15) | X | ✓ | ✓ | ✓ | ✓ | ✓ | ✓ | ✓ | ✓ | ✓ | Low |
| Lin et al. 2005 (16) | ✓ | X | ✓ | ✓ | ✓ | ✓ | ✓ | ✓ | ✓ | X | Low |
| Lin & Shah 2008 (17) | ✓ | X | ✓ | ✓ | ✓ | ✓ | X | ✓ | ✓ | X | Moderate |
| Lin et al. 2008 (18) | X | X | ✓ | ✓ | ✓ | ✓ | X | ✓ | ✓ | X | Moderate |
| Ma et al. 2014 (19) | ✓ | ✓ | ✓ | ✓ | ✓ | ✓ | X | ✓ | ✓ | ✓ | Low |
| Manivannan et al. 2014 (20) | X | X | ✓ | ✓ | ✓ | ✓ | ✓ | ✓ | ✓ | ✓ | Low |
| McIntyre et al. 2005 (21) | X | X | X | X | X | ✓ | ✓ | ✓ | ✓ | ✓ | High |
| Moro Moro et al. 2011 (22) | X | X | ✓ | ✓ | ✓ | ✓ | ✓ | ✓ | ✓ | ✓ | Low |
| Motosue et al. 2017 (23) | X | X | ✓ | ✓ | ✓ | ✓ | ✓ | ✓ | ✓ | X | Moderate |
| Mulla & Simon 2007 (24) | ✓ | X | ✓ | ✓ | ✓ | ✓ | X | ✓ | ✓ | X | Moderate |
| Mulla & Simons 2013 (25) | ✓ | X | X | ✓ | ✓ | ✓ | X | ✓ | ✓ | ✓ | Moderate |
| Mullins 2003 (26) | X | X | X | X | ✓ | ✓ | X | ✓ | X | ✓ | High |
| Mullins 2007 (27) | X | X | ✓ | ✓ | ✓ | ✓ | X | ✓ | ✓ | X | Moderate |
| Mullins et al. 2009 (28) | X | X | ✓ | ✓ | ✓ | ✓ | X | ✓ | ✓ | X | Moderate |
| Mullins et al. 2015 (29) | ✓ | X | ✓ | ✓ | ✓ | ✓ | X | ✓ | ✓ | X | Moderate |
| Mullins et al. 2016 (30) | ✓ | X | ✓ | ✓ | ✓ | ✓ | X | ✓ | ✓ | X | Moderate |
| Norredam et al. 2016 (31) | ✓ | ✓ | ✓ | ✓ | ✓ | ✓ | X | ✓ | ✓ | X | Low |
| Poulos et al. 2007 (32) | ✓ | X | ✓ | ✓ | ✓ | ✓ | X | ✓ | ✓ | X | Moderate |
| Pourang et al. 2017 (33) | X | ✓ | ✓ | ✓ | ✓ | ✓ | ✓ | ✓ | ✓ | ✓ | Low |
| Simons et al. 2002 (34) | X | X | ✓ | ✓ | ✓ | ✓ | X | ✓ | ✓ | X | Moderate |
| Smit et al. 2005 (35) | X | X | ✓ | ✓ | ✓ | ✓ | ✓ | ✓ | ✓ | ✓ | Low |
| Stewart & Ewan 1996 (36) | X | X | ✓ | ✓ | ✓ | ✓ | ✓ | ✓ | ✓ | ✓ | Low |
| Tanno et al. 2017 (37) | X | X | ✓ | ✓ | ✓ | ✓ | ✓ | ✓ | ✓ | ✓ | Low |
| Tejedor Alonso et al. 2011 (38) | X | X | ✓ | ✓ | ✓ | ✓ | ✓ | ✓ | ✓ | ✓ | Low |
| Tejedor Alonso et al. 2012 (39) | ✓ | ✓ | ✓ | ✓ | ✓ | ✓ | ✓ | ✓ | ✓ | ✓ | Low |
| Tejedor Alonso et al. 2015 (40) | ✓ | X | ✓ | ✓ | ✓ | ✓ | ✓ | ✓ | ✓ | ✓ | Low |
| Tham et al. 2008 (41) | ✓ | X | ✓ | ✓ | ✓ | ✓ | X | ✓ | ✓ | ✓ | Low |
| Turner et al. 2015 (42) | ✓ | X | ✓ | ✓ | ✓ | ✓ | X | ✓ | ✓ | X | Moderate |
| Van der Klauw et al. 1993 (43) | ✓ | X | ✓ | X | ✓ | ✓ | ✓ | ✓ | ✓ | X | Moderate |
| Yang et al. 2017 (44) | ✓ | ✓ | ✓ | ✓ | ✓ | ✓ | X | ✓ | ✓ | X | Low |
| Yocum et al. 1999 (45) | X | ✓ | ✓ | ✓ | ✓ | ✓ | ✓ | ✓ | ✓ | X | Low |
| Yoon et al. 2017 (46) | X | ✓ | ✓ | ✓ | ✓ | ✓ | ✓ | ✓ | ✓ | X | Low |

Risk of bias assessment questions: ✓ (low risk of bias), X (high risk of bias)

Questions applied to assess all included articles:

External validity

Q1: Was the study’s target population a close representation of the national population in relation to relevant variables?

Q2: Was the sampling frame a true or close representation of the target population?

Q3: Was some form of random selection used to select the sample, OR was a census undertaken?

Q4: Was the likelihood of nonresponse bias minimal?

Internal validity

Q5: Were data collected directly from the subjects (as opposed to a proxy)?

Q6: Was an acceptable case definition used in the study?

Q7: Was the study instrument that measured the parameter of interest shown to have validity and reliability?

Q8: Was the same mode of data collection used for all subjects?

Q9: Was the length of the shortest prevalence period for the parameter of interest appropriate?

Q10: Were the numerator(s) and denominator(s) for the parameter of interest appropriate?

Summary item on the overall risk of study bias:

Low risk of bias: 8 or more “✓” answers; Moderate risk of bias: 6 to 7 “✓” answers; High risk of bias: 5 or fewer “✓” answers

## Supplementary Table 6. Original articles concerning all-cause anaphylaxis together with their period and country of analysis and the reported incidence per 100,000 population per year.

| **Publication** | **Period of analysis** | **Country** | **Size of reference population** | **Data source** | **Incidence [per 100,000 population per year]** |
| --- | --- | --- | --- | --- | --- |
| Yoon et al. 2017 (46) | 2009 - 2014 | Korea region | 6,000,000 | Hospital attendances | 0.49 |
| Cetinkaya et al. 2013 (6) | 2010 - 2011 | Istanbul (Turkey) | 12,703,000 (47) | Admissions | 0.90 |
| Lin et al. 2008 (18) | 1990 | New York State | 5,523,070 (48) † | Hospitalizations | 1.0 |
| Lin & Shah 2008 (17) | 2001 | U.S. | 285,081,556 (49) | Hospitalizations | 1.99 |
| Lin & Shah 2008 (17) | 2003 | U.S. | 290,326,418 (49) | Hospitalizations | 2.00 |
| Lin & Shah 2008 (17) | 2002 | U.S. | 287,803,914 (49) | Hospitalizations | 2.05 |
| Lin & Shah 2008 (17) | 1998 | U.S. | 270,248,000 (49) | Hospitalizations | 2.07 |
| Lin & Shah 2008 (17) | 2000 | U.S. | 282,171,957 (49) | Hospitalizations | 2.10 |
| Lin et al. 2005 (16) | 1990 - 2003 | New York State | 18,976,457 (50) | Hospitalizations | 2.11 |
| Lin & Shah 2008 (17) | 1999 | U.S. | 272,691,000 (49) | Hospitalizations | 2.15 |
| Lin & Shah 2008 (17) | 2004 | U.S. | 293,045,739 (49) | Hospitalizations | 2.17 |
| Lin & Shah 2008 (17) | 2005 | U.S. | 295,753,151 (49) | Hospitalizations | 2.26 |
| Lin et al. 2008 (18) | 2006 | New York State | 5,094,314 (51) † | Hospitalizations | 4.7 |
| Turner et al. 2015 (42) | 1992 | England and Wales | 52,360,000 (52) | Hospital admissions | 1.00 |
| Turner et al. 2015 (42) | 2012 | England and Wales | 52,360,000 (52) | Hospital admissions | 7.00 |
| Van der Klauw et al. 1993 (43) | 1987 - 1988 | Netherlands | 14,679,077 (53) | Admissions | 1.14 |
| Tham et al. 2008 (41) | 1999 - 2004 | Singapore | 4,088,336 | Epinephrine prescription (auto-injectable) | 1.20 |
| Tejedor Alonso et al. 2015 (40) | 1998 | Spain | 39,852,649 | Admissions | 1.32 |
| Tejedor Alonso et al. 2015 (40) | 2000 | Spain | 40,499,789 | Admissions | 1.40 |
| Tejedor Alonso et al. 2015 (40) | 1999 | Spain | 40,202,159 | Admissions | 1.42 |
| Tejedor Alonso et al. 2015 (40) | 2001 | Spain | 41,116,841 | Admissions | 1.53 |
| Tejedor Alonso et al. 2015 (40) | 2002 | Spain | 41,837,895 | Admissions | 1.54 |
| Tejedor Alonso et al. 2015 (40) | 2003 | Spain | 42,717,064 | Admissions | 1.60 |
| Tejedor Alonso et al. 2015 (40) | 2004 | Spain | 43,197,684 | Admissions | 1.66 |
| Tejedor Alonso et al. 2011 (38) | 1999 - 2005 | Madrid (Spain) | 250,000 | Admissions | 1.70 |
| Tejedor Alonso et al. 2015 (40) | 2005 | Spain | 44,108,530 | Admissions | 1.71 |
| Tejedor Alonso et al. 2015 (40) | 2007 | Spain | 45,200,737 | Admissions | 1.71 |
| Tejedor Alonso et al. 2015 (40) | 2006 | Spain | 44,708,964 | Admissions | 1.81 |
| Tejedor Alonso et al. 2015 (40) | 2008 | Spain | 46,157,822 | Admissions | 1.89 |
| Tejedor Alonso et al. 2015 (40) | 2009 | Spain | 46,745,807 | Admissions | 2.08 |
| Tejedor Alonso et al. 2015 (40) | 2011 | Spain | 47,190,493 | Admissions | 2.50 |
| Tejedor Alonso et al. 2015 (40) | 2010 | Spain | 47,021,031 | Admissions | 2.51 |
| Tejedor Alonso et al. 2012 (39) | 2005 | Alcorcon (Spain) | 150,000 | Multiple clinical  settings | 104.00 |
| Tejedor Alonso et al. 2012 (39) | 2004 | Alcorcon (Spain) | 150,000 | Multiple clinical  settings | 120.00 |
| Hoyos-Bachiloglu et al. 2014 (12) | 2001 - 2010 | Chile | 16,425,532 | Admissions | 1.41 |
| Beyer et al. 2012 (2) | 2009 | Berlin (Germany) | 3,442,675 | Emergency  physicians | 1.63 |
| Beyer et al. 2012 (2) | 2010 | Berlin (Germany) | 3,460,725 | Emergency  physicians | 2.17 |
| Beyer et al. 2012 (2) | 2008 | Berlin (Germany) | 3,431,675 | Emergency  physicians | 4.52 |
| Stewart & Ewan 1996 (36) | 1993 | Cambridge (U.K.) | 350,000 | Accident & emergency department | 2.57 |
| Stewart & Ewan 1996 (36) | 1994 | Cambridge (U.K.) | 350,000 | Accident & emergency  department | 10.29 |
| Mulla & Simons 2013 (25) | 2004 - 2007 | Texas (U.S.) | 22,778,123 | Inpatients | 2.65 |
| Mulla & Simon 2007 (24) | 2001 | Florida (U.S.) | 16,410,669 | Hospitalizations | 2.69 |
| Mullins 2007 (27) | 1993 - 1994 | Australia | 17,760,916 (54) | Hospital admissions | 3.62 |
| Mullins et al. 2016 (30) | 1997 | Australia | 18,467,588 (55) | Hospital admissions | 5 |
| Mullins et al. 2015 (29) | 1998 - 1999 | Australia | 18,906,936 (55) | Hospital admissions | 6.3 |
| Mullins 2007 (27) | 2004 - 2005 | Australia | 20,210,057 (54) | Hospital admissions | 8.03 |
| Mullins et al. 2015 (29) | 2004 - 2005 | Australia | 20,274,282 (55) | Hospital admissions | 10.6 |
| Mullins et al. 2015 (29) | 2005 - 2006 | Australia | 20,606,228 (55) | Hospital admissions | 12.2 |
| Mullins 2003 (26) | 1995 - 2000 | Australian Capital Territory | 330,000 | Specialist medical practices | 12.89 |
| Mullins et al. 2009 (28) | 2002 - 2007 | Australian regions | 15,057,146 (55) | Hospital admissions | 14.60 |
| Mullins et al. 2015 (29) | 2011 - 2012 | Australia | 22,911,375 (55) | Hospital admissions | 17.7 |
| Mullins et al. 2016 (30) | 2013 | Australia | 23,150,729 (55) | Hospital admissions | 19.2 |
| Poulos et al. 2007 (32) | 1993 - 1994 | Australia | 19,308,681 (55) | Hospital admissions /  hospitalizations | 3.7 |
| Poulos et al. 2007 (32) | 2004 - 2005 | Australia | 19,308,681 (55) | Hospital admissions / hospitalizations | 10.8 |
| Gaeta et al. 2007 (8) | 1993 - 2004 | U.S. | 296,650,718 | ED | 3.80 |
| Calvani et al. 2008 (5) | 2001 | Lazio (Italy) | 861,062 † | EDs, hospitalizations and day hospitals | 3.95 |
| Calvani et al. 2008 (5) | 2003 | Lazio (Italy) | 866,111 † | EDs, hospitalizations and day hospitals | 6.12 |
| Calvani et al. 2008 (5) | 2002 | Lazio (Italy) | 860,046 † | EDs, hospitalizations and day hospitals | 6.40 |
| Calvani et al. 2008 (5) | 2000 | Lazio (Italy) | 866,989 † | EDs, hospitalizations and day hospitals | 7.04 |
| Clark et al. 2014 (7) | 2002 - 2008 | U.S. | 43,000,000 | EDs and hospitals | 3.98 |
| Smit et al. 2005 (35) | 1999 - 2003 | Hong Kong | 1,500,000 | ED | 4.70 |
| Kivistö et al. 2016 (13) | 1999 - 2011 | Finland | 1,248,284 † | Hospitalizations | 6.03 |
| Kivistö et al. 2016 (13) | 1999 - 2011 | Sweden | 2,164,270 † | Hospitalizations | 7.76 |
| McIntyre et al. 2005 (21) | 2001 - 2003 | Massachu­setts (U.S.) | 798,762 | Epinephrine  administration | 6.89 |
| Norredam et al. 2016 (31) | 1994 - 2010 | Denmark | 867,850 | Hospital attendances  (first time) | 7.14 |
| Hananashvili et al. 2016 (9) | 2008 | Negev (Israel) | 326,340 ‡ | ED and hospitalizations | 7.35 |
| Hananashvili et al. 2016 (9) | 2010 | Negev (Israel) | 357,640 ‡ | ED and hospitalizations | 7.83 |
| Hananashvili et al. 2016 (9) | 2009 | Negev (Israel) | 350,840 ‡ | ED and hospitalizations | 8.55 |
| Hananashvili et al. 2016 (9) | 2011 | Negev (Israel) | 364,040 ‡ | ED and hospitalizations | 21.15 |
| Hananashvili et al. 2016 (9) | 2012 | Negev (Israel) | 364,040 | ED and hospitalizations | 36.26 |
| Harduar-Morano et al. 2011 (10) | 2005 - 2006 | Florida (U.S.) | 18,057,130 (56) | EDs | 7.62 |
| Helbling et al. 2004 (11) | 1996 | Canton Bern (Switzerland) | 940,000 | Allergy clinics,  specialists and EDs | 7.87 |
| Helbling et al. 2004 (11) | 1997 | Canton Bern (Switzerland) | 940,000 | Allergy clinics,  specialists and EDs | 9.15 |
| Helbling et al. 2004 (11) | 1998 | Canton Bern (Switzerland) | 940,000 | Allergy clinics,  specialists and EDs | 9.79 |
| Ma et al. 2014 (19) | 2007 | U.S. | 301,600,000 | EDs and hospitalizations | 8.22 |
| Ma et al. 2014 (19) | 2006 | U.S. | 304,100,000 | EDs and hospitalizations | 8.46 |
| Ma et al. 2014 (19) | 2008 | U.S. | 299,400,000 | EDs and hospitalizations | 9.32 |
| Ma et al. 2014 (19) | 2009 | U.S. | 307,000,000 | EDs and hospitalizations | 9.62 |
| Manivannan et al. 2014 (20) | 2002 - 2007 | not reported | 130,000 | Emergency transports | 9.69 |
| Lauritano et al. 2013 (14) | 2009 - 2010 | Alessandria (Italy) | 150,000 ‡ | ED | 12.00 |
| Motosue et al. 2017 (23) | 2005 | U.S. | 24,802,817 | EDs | 14.2 |
| Motosue et al. 2017 (23) | 2006 | U.S. | 26,006,369 | EDs | 15.7 |
| Motosue et al. 2017 (23) | 2007 | U.S. | 25,727,273 | EDs | 17.6 |
| Motosue et al. 2017 (23) | 2008 | U.S. | 25,201,058 | EDs | 18.9 |
| Motosue et al. 2017 (23) | 2009 | U.S. | 24,085,308 | EDs | 21.1 |
| Motosue et al. 2017 (23) | 2010 | U.S. | 24,039,648 | EDs | 22.7 |
| Motosue et al. 2017 (23) | 2011 | U.S. | 25,080,000 | EDs | 25.0 |
| Motosue et al. 2017 (23) | 2012 | U.S. | 26,389,892 | EDs | 27.7 |
| Motosue et al. 2017 (23) | 2013 | U.S. | 27,208,481 | EDs | 28.3 |
| Motosue et al. 2017 (23) | 2014 | U.S. | 26,213,287 | EDs | 28.6 |
| Yang et al. 2017 (44) | 2008 | Korea | 49,218,985 (57) | All care providers | 15.68 |
| Yang et al. 2017 (44) | 2009 | Korea | 49,379,208 (57) | All care providers | 17.62 |
| Yang et al. 2017 (44) | 2010 | Korea | 49,552,855 (57) | All care providers | 19.16 |
| Yang et al. 2017 (44) | 2011 | Korea | 49,744,659 (57) | All care providers | 19.47 |
| Yang et al. 2017 (44) | 2012 | Korea | 49,952,244 (57) | All care providers | 23.18 |
| Yang et al. 2017 (44) | 2013 | Korea | 50,169,242 (57) | All care providers | 25.00 |
| Yang et al. 2017 (44) | 2014 | Korea | 50,385,560 (57) | All care providers | 32.15 |
| Lee et al. 2017 (15) | 2005 | Olmsted County (U.S.) | 134,953 (58) | All care providers | 20.75 |
| Lee et al. 2017 (15) | 2004 | Olmsted County (U.S.) | 133,195 (58) | All care providers | 36.04 |
| Lee et al. 2017 (15) | 2001 | Olmsted County (U.S.) | 126,737 (58) | All care providers | 42.61 |
| Lee et al. 2017 (15) | 2006 | Olmsted County (U.S.) | 137,293 (58) | All care providers | 43.70 |
| Lee et al. 2017 (15) | 2003 | Olmsted County (U.S.) | 131,196 (58) | All care providers | 46.50 |
| Lee et al. 2017 (15) | 2010 | Olmsted County (U.S.) | 144,248 (58) | All care providers | 49.22 |
| Lee et al. 2017 (15) | 2002 | Olmsted County (U.S.) | 129,132 (58) | All care providers | 49.56 |
| Lee et al. 2017 (15) | 2008 | Olmsted County (U.S.) | 141,527 (58) | All care providers | 52.29 |
| Lee et al. 2017 (15) | 2007 | Olmsted County (U.S.) | 139,369 (58) | All care providers | 59.55 |
| Lee et al. 2017 (15) | 2009 | Olmsted County (U.S.) | 143,521 (58) | All care providers | 61.32 |
| Brown et al. 2001 (3) | 1998 - 1999 | Brisbane  (Australia) | 485,000 ‡ | ED | 29.28 |
| Yocum et al. 1999 (45) | 1983 - 1987 | Olmsted County (U.S.) | 97,934 (59) | All care providers | 31.45 |
| Tanno et al. 2017 (37) | 2014 | Montpellier (France) | 270,000 | Inpatients  (direct referrals) | 32.96 |
| Moro Moro et al. 2011 (22) | 2004 - 2005 | Madrid (Spain) | 250,000 | ED | 42.60 |
| Buka et al. 2015 (4) | 2012 | U.K. region | 891,159 | EDs | 47.8 |
| Anandan et al. 2009 (1) | 2004 - 2005 | Scotland | 5,094,800 (60) | Admissions /  Inpatient stays | 52.00 |
| Simons et al. 2002 (34) | 1995 - 2002 | Manitoba (Canada) | 1,147,694 | Epinephrine  prescription | 190.80 |
| Pourang et al. 2017 (33) | 2008 | Southern California (U.S.) | 2.531.563 | Health maintenance organization | 307.9 |
| Pourang et al. 2017 (33) | 2009 | Southern California (U.S.) | 2.531.563 | Health maintenance organization | 308.4 |
| Pourang et al. 2017 (33) | 2011 | Southern California (U.S.) | 2.531.563 | Health maintenance organization | 309.9 |
| Pourang et al. 2017 (33) | 2010 | Southern California (U.S.) | 2.531.563 | Health maintenance organization | 315.4 |
| Pourang et al. 2017 (33) | 2012 | Southern California (U.S.) | 2.531.563 | Health maintenance organization | 328.7 |

Supplementary Table 6 includes all publications that reported population based all-cause anaphylaxis incidence rates. The rows were ranked primarily based on the incidence values in increasing order; thus rows concerning the same first author were grouped together. When equally high incidence rates were noted, the rows were ranked based on the respective periods of analysis.

ED: Emergency department.

† reference population pediatric; ‡ reference population adult.

## Supplementary Table 7. Overview of anaphylaxis definitions used in original articles taken for this study. The articles are ordered based on the definition of anaphylaxis starting with ICD codes, followed by text strings, a combination of code/text search and review of patient records, review of patient records based on recommended criteria, review of patient records based on self-defined criteria and finally epinephrine prescriptions.

| **Publication** | **Definition of anaphylaxis** |
| --- | --- |
| Gaeta et al. 2007 (8) | ICD-9 codes: 995.0, 995.6 |
| Lin et al. 2008 (18) | ICD-9-CM codes: 995.0, 995.6, 999.4 |
| Lin & Shah 2008 (17) |  |
| Lin et al. 2005 (16) |  |
| Ma et al. 2014 (19) |  |
| Mulla & Simon 2007 (24) | ICD-9-CM codes: 989.5 (unless additional code E905.0, E905.1, E905.2, E905.4, E905.6, E905.7),  995.0, 995.4, 995.60 – 995.69 |
| Calvani et al. 2008 (5) | ICD-9-CM codes: 995.0, 995.4, 995.60 – 995.69, 999.4 |
| Mulla & Simons 2013 (25) | ICD-9-CM codes: 995, 995.4, 995.60 – 995.69, 999.4 |
| Pourang et al. 2017 (33) | ICD-9 codes: 989.5, 995.0, 995.1, 995.4, 995.60 – 995.69, E905.3, E905.5, E905.8, E905.9 |
| Turner et al. 2015 (42) | ICD-9 codes: 995.0, 995.6  ICD-10 codes: T78.0, T78.2, T88.6 |
| Mullins 2007 (27) | ICD-9 codes: 995.0, 995.6, 999.4  ICD-10 codes: T78, T78.2, T80.5 |
| Poulos et al. 2007 (32) | ICD-9-CM codes: 995.0, 995.6, 999.4  ICD-10-AM codes: T78.0, T78.2, T80.5, T88.6 |
| Mullins et al. 2016 (30) | ICD-9 codes: 995.0, 995.6, 999.4  ICD-10 codes: T78, T78.2, T80.5, T88.6 |
| Kivistö et al. 2016 (13) | ICD-10 codes: 78.0, 78.2 |
| Hoyos-Bachiloglu et al. 2014 (12) | ICD-10 codes: T78.0, T78.2, T88.6 |
| Mullins et al. 2015 (29) | ICD-10 codes: T78, T78.2, T80.5, T88.6 |
| Mullins et al. 2009 (28) |  |
| Anandan et al. 2009 (1) | ICD-10 codes: T78.0, T78.2, T80.5, T88.6 |
| Yang et al. 2017 (44) |  |
| Norredam et al. 2016 (31) | ICD-10 codes: T63.4F, T78.0, T78.2, T80.5, T88.6 |
| Cetinkaya et al. 2013 (6) | ICD-10-CM:  one code specific for anaphylaxis (T78.0, T78.2, T80.5, T88.6), or  at least two codes with signs or symptoms of anaphylaxis (I95.2, I95.0, L27.0, L50.0, L50.1, L50.9, R06.0, R06.2, R09.0, R09.2, R11, R55, R57, R60.1) |
| Clark et al. 2014 (7) | ICD-9-CM codes: validated algorithm by Harduar-Morano et al. (61) |
| Harduar-Morano et al. 2011 (10) |  |
| Motosue et al. 2017 (23) |  |
| Tejedor Alonso et al. 2015 (40) | ICD-9-CM codes adapted from validated algorithm by Harduar-Morano et al. (61) |
| Mullins 2003 (26) | ICD-9 codes: 995.0, 995.4, 995.6, 999.4;  Then review of patient records based on the following criteria:  “two or more of urticaria/angioedema, bronchospasm, gastrointestinal symptoms or hypotension” (62) |
| Tejedor Alonso et al. 2011 (38) | ICD-9-CM (Sixth Edition) codes: 708, 782.3, 782.62, 785.50, 785.59, 995.0, 995.1, 995.2, 995.3, 995.4, 995.6, 998.0, 999.4, 999.5, 999.8, E879.*, E905.3, E930 – E949, V14.0, V15.01 – V15.05, V15.06, V15.07, V15.08, V15.09;  Then review of patient records based on NIAID/FAAN anaphylaxis criteria (63) |
| Lee et al. 2017 (15) | ICD-9 codes:  sublist A: 995.0, 995.60 – 995.69, 999.41 – 999.42, 999.49, V13.81  sublist B: 989.5, E905.0 – E905.9, V15.06,  sublist C: 477.1, 692.5, 693.1, 988.0 – 988.2, 988.8 – 988.9, 995.7, V15.01 – V15.05,  sublist D: 995.20 – 995.21, 995.27  Then review of all patient records of sublist A and 20% of sublists B – D based on NIAID/FAAN anaphylaxis criteria (63) |
| Moro Moro et al. 2011 (22) | Database search for ‘alerg’/‘alergi*’, ‘anafila’/‘anafila*’, ‘urtica’/‘urtica*’, ‘hipersensibili’/‘hipersensibili*’, ‘eritema’/ ‘eritema*’, ‘picadu’/‘picadu*’, ‘advers’/‘advers*’, ‘edem’/‘edem*’, ‘medica’/‘medica*’, ‘reacc’/‘reacc*’, ‘alimen’/‘alimen*’, ‘abeja’/‘abeja*’, ‘avispa’/‘avispa*’;  Then review of patient records based on NIAID/FAAN anaphylaxis criteria (63) |
| Tejedor Alonso et al. 2012 (39) |  |
| Hananashvili et al. 2016 (9) | Database search for ‘anaphylactic shock or reaction’ and ‘allergic reaction’;  Then review of patient records based on NIAID/FAAN anaphylaxis criteria (63) |
| Manivannan et al. 2014 (20) | Database search for ‘allergic reaction’, ‘animal bite/sting’, ‘anaphylaxis’, ‘swollen tongue’, ‘bronchospasm’, ‘unresponsive’, ‘hypotension’, ‘wheezing’, ‘environmental’, ‘dyspnea’, ‘rash’, ‘drug reaction’;  Then review of patient records based on NIAID/FAAN anaphylaxis criteria [modified from Sampson et al. (63)] |
| Yoon et al. 2017 (46) | ICD-10: codes for “anaphylaxis, anaphylaxis shock, allergy and anaphylaxis, hypersensitivity, food hypersensitivity, and drug hypersensitivity”;  Then review of patient records based on the following criteria:  “respiratory or cardiovascular symptoms accompanied by an acute cutaneous reaction within a few minutes to a few hours”, or  “two or more systemic responses out of the cutaneous, respiratory, cardiovascular, or gastrointestinal system, after exposure to an allergic antigen”, or  “hypotension after exposure to a known allergic antigen” (63) |
| Buka et al. 2015 (4) | WAO anaphylaxis guidelines (64) |
| Smit et al. 2005 (35) | Terms in database: “allergy, allergic reaction, anaphylactic reaction or anaphylactic shock, anaphylaxis, anaphylactoid reaction, bee stings, insect bites, drug reactions, angioedema / angioneurotic edema, or urticaria”  Anaphylaxis criteria:  “hypotension, severe cutaneous manifestation, respiratory or airway compromise, cardiovascular compromise such as hypotension or dysrhythmias, syncope or loss of consciousness, or any suspicion […] of likely respiratory or circulatory compromise.”; |
| Lauritano et al. 2013 (14) | Severe anaphylaxis:  ICD-9-CM codes: 493.9, 708.0, 995.0, 995.1, 995.3;  Then review of patient records based on the following criteria: “severe, life-threatening, generalized or systemic hypersensitivity reaction rapidly developing life-threatening airway and/or breathing and/or circulation problems” |
| Brown et al. 2001 (3) | ICD-9-CM:  16 codes under 4 major headings: allergy/allergic reaction, anaphylactic shock or reaction, angioedema, urticaria;  Then review of patient records based on the following criteria: “Mild to moderate anaphylaxis: Patients with any of the findings listed for acute allergic reaction” (i.e. “Patients with evidence of generalized mediator release restricted to cutaneous findings alone, such as generalized rash, pruritus, rhinitis/conjunctivitis, urticaria, local edema, and angioedema without any other systemic symptoms or signs”) “with additional respiratory, cardiovascular, gastrointestinal, or neurologic features or presenting with any of these additional features alone in the setting of an allergic reaction. The additional features included a history of shortness of breath or dyspnea, wheeze, hoarseness, and nausea or vomiting. Physical findings recorded on arrival in the ED or within 30 minutes of arrival included the presence of bronchospasm, systolic blood pressure >90 mmHg, respiratory rate <25/min, and a normal Glasgow Coma Scale score.” |
| Yocum et al. 1999 (45) | Database search for ‘anaphylaxis’, ‘food allergy’, ‘drug allergy’, ‘hypersensitivity not otherwise specified’, ‘adverse effect of immunotherapy’, ‘adverse effect of injectable diagnostic agent’, ‘subglottic or pharyngeal edema’, ‘bee sting allergy’, ‘unspecified allergic reaction’;  Then review of patient records based on the following criteria: “1 symptom of generalized mediator release, such as flushing; pruritus or paresthesias of lips, axilla, hands, or feet; general pruritus; urticaria or angioedema; lip tingling or paresthesia; and conjunctivitis or chemosis. In addition to 1 symptom of generalized mediator release, we required at least 1 of the following additional symptoms to be present during the event, involving the oral and gastrointestinal, respiratory, or cardiovascular system, as discussed below.  1. Oral and gastrointestinal: oral mucosal pruritus; intraoral angioedema of buccal mucosa, tongue, palate, or oropharynx; nausea, emesis, dysphagia, abdominal cramps, or diarrhea.  2. Respiratory: rhinitis, stridor, cough, hoarseness, aphonia, tightness in the throat, dyspnea, wheezing, hypopharyngeal or laryngeal edema, cyanosis.  3. Cardiovascular: chest pain, arrhythmia, hypotension, presyncope, syncope, tachycardia, bradycardia, orthostasis, seizures, and shock. Only 2 exceptions to these criteria could classify an event as anaphylaxis: isolated laryngeal edema or immediate shock and a syncopal event after injection of medication or a radiocontrast agent.” |
| Tanno et al. 2017 (37) | ICD-10 codes (2015 version): T78.0 – T78.8, T80.5;  Then review of patient records by exclusion of:  allergic or hypersensitivity comorbidities, or  “(i) descriptions resembling allergic or hypersensitivity conditions, but not considered as real allergies or hypersensitivities (e.g. food intolerance or Munchausen syndrome); (ii) transversal analysis in which the description of allergic or hypersensitivity conditions was reported before or after the time of the evaluation, and  (iii) unclear described diagnosis (e.g. files containing initial hypothesis of anaphylaxis, but clinical evaluation and management of an episode of isolated bronchospasm).” |
| Van der Klauw et al. 1993 (43) | ICD-9-CM:  all with codes 989.5, 995.0, 995.4, 999.4,  random sample with codes 693.0, 695.0, 708.0, 995.1, 995.2, 995.3;  Then review of patient records based on causality:  anaphylaxis probable: „symptoms characteristic of anaphylaxis, i.e. symptoms out of two or more of the following four systems (with the exception of a combination of system 1 and 4) and if the reaction had occurred within 1 h after exposure to the causative agent (or had been specified as ‘shortly’ or ‘immediately’ after exposure): 1. Cardiovascular system: collapse, loss of consciousness, hypotension (systolic blood pressure – 100 mm Hg and symptoms characteristic of hypotension or, in case of hypertension, a blood pressure that gave symptoms characteristic of hypotension). 2. Respiratory system: rhinitis, swelling of the uvula or pharynx, laryngeal edema (stridor), bronchospasm (dyspnea, wheezing, asthma). 3. Skin and conjunctiva: pruritus, erythema, urticaria, angioedema, conjunctivitis. 4. Gastrointestinal system: nausea, vomiting, diarrhea, fecal urge, abdominal pain or spasm“,  anaphylaxis possible: „symptoms compatible with – but not characteristic of – anaphylaxis, i.e. symptoms out of only one of the systems 1, 2 or 3 or the combination of system 1 and 4“ and „occurred within 1 h or if the time interval between exposure and reaction was unknown“ or „if the symptoms were characteristic of anaphylaxis, but the reaction had occurred later than an hour after exposure or this time period was unknown“ |
| Stewart & Ewan 1996 (36) | Database search for  diagnosis of allergy or sting, or  coding of ‘skin and subcutaneous’ or ‘collapse’;  Then review of patient records |
| Beyer et al. 2012 (2) | Severe anaphylaxis:  severe respiratory symptoms “(dyspnoea, stridor, apnoea)” (level 1), or  severe cardiovascular symptoms “(decreased alertness, tachycardia, decrease in blood pressure, loss of consciousness, collapse, cardiac arrest)” (level 2), or respiratory and cardiovascular symptoms (level 3) |
| Helbling et al. 2004 (11) | “Severe systemic anaphylaxis with circulatory symptoms (hypotension, unconsciousness, or shock) consistent with a generalized mast cell-mediated reaction”:  “Symptoms considered as immediate usually occurred within a period of 2 h after contact, ingestion, or parenteral injection of a potential allergen or suspected agent. The diagnosis of anaphylaxis was set after an allergological workup based on history, consistent symptoms and signs of a mast cell release, and allergy testing. Patients with isolated asthmatic symptoms were not included.” |
| McIntyre et al. 2005 (21) | Epinephrine prescriptions |
| Simons et al. 2002 (34) |  |
| Tham et al. 2008 (41) | Epinephrine auto-injector prescriptions |

# Supplementary Figures

**Continents**

Incidence
with 95% CI

17.22 [ 10.55, 28.13 ]

71.16 [ 22.50, 225.00 ]

43.23 [ 20.24, 92.33 ]

7.60 [ 2.87, 20.13 ]

## Supplementary Figure 1. Forest plot illustrating pooled incidence rates (in cases per 100,000 population per year) per continent. The results of South America are not shown since it concerns only one included article. Moreover, the study not reporting the continent of analysis was excluded from this graph.

53.23 [ 28.51, 99.39 ]

5.23 [ 3.38, 8.11 ]

19.6 [ 13.13, 29.27 ]

**Age Groups**

Incidence
with 95% CI

**Age groups**

## Supplementary Figure 2. Forest plot illustrating pooled incidence rates (in cases per 100,000 population per year) of articles that did not restrict their analysis on particular age groups (‘all’), as well as those that analyze the pediatric population only or the adult population only (‘non-pediatric’).

# Supplementary References

1. Anandan C, Gupta R, Simpson CR, Fischbacher C, Sheikh A. Epidemiology and disease burden from allergic disease in Scotland: Analyses of national databases. *J R Soc Med* (2009) **102**:431–42. doi:10.1258/jrsm.2009.090027

2. Beyer K, Eckermann O, Hompes S, Grabenhenrich L, Worm M. Anaphylaxis in an emergency setting - elicitors, therapy and incidence of severe allergic reactions. *Allergy* (2012) **67**:1451–6. doi:10.1111/all.12012

3. Brown AF, McKinnon D, Chu K. Emergency department anaphylaxis: A review of 142 patients in a single year. *J Allergy Clin Immunol* (2001) **108**:861–6. doi:10.1067/mai.2001.119028

4. Buka RJ, Crossman RJ, Melchior CL, Huissoon AP, Hackett S, Dorrian S, et al. Anaphylaxis and ethnicity: Higher incidence in British South Asians. *Allergy* (2015) **70**:1580–7. doi:10.1111/all.12702

5. Calvani M, Di Lallo D, Polo A, Spinelli A, Zappalà D, Zicari AM. Hospitalizations for pediatric anaphylaxis. *Int J Immunopathol Pharmacol* (2008) **21**:977–83. doi:10.1177/039463200802100422

6. Cetinkaya F, Incioglu A, Birinci S, Karaman BE, Dokucu AI, Sheikh A. Hospital admissions for anaphylaxis in Istanbul, Turkey. *Allergy* (2013) **68**:128–30. doi:10.1111/all.12069

7. Clark S, Wei W, Rudders SA, Camargo CA. Risk factors for severe anaphylaxis in patients receiving anaphylaxis treatment in US emergency departments and hospitals. *J Allergy Clin Immunol* (2014) **134**:1125–30. doi:10.1016/j.jaci.2014.05.018

8. Gaeta TJ, Clark S, Pelletier AJ, Camargo CA. National study of US emergency department visits for acute allergic reactions, 1993 to 2004. *Ann Allergy Asthma Immunol* (2007) **98**:360–5. doi:10.1016/S1081-1206(10)60883-6

9. Hananashvili I, Givon-Lavi N, Bartal C, Broides A. Anaphylactic reactions in adult patients in Southern Israel. *Asian Pac J Allergy Immunol* (2016) **34**:44–50. doi:10.12932/AP0521.34.1.2016

10. Harduar-Morano L, Simon MR, Watkins S, Blackmore C. A population-based epidemiologic study of emergency department visits for anaphylaxis in Florida. *J Allergy Clin Immunol* (2011) **128**:594-600.e1. doi:10.1016/j.jaci.2011.04.049

11. Helbling A, Hurni T, Mueller UR, Pichler WJ. Incidence of anaphylaxis with circulatory symptoms: A study over a 3-year period comprising 940,000 inhabitants of the Swiss Canton Bern. *Clin Exp Allergy* (2004) **34**:285–90.

12. Hoyos-Bachiloglu R, Morales PS, Cerda J, Talesnik E, González G, Camargo CA, et al. Higher latitude and lower solar radiation influence on anaphylaxis in Chilean children. *Pediatr Allergy Immunol* (2014) **25**:338–43. doi:10.1111/pai.12211

13. Kivistö JE, Protudjer JL, Karjalainen J, Wickman M, Bergström A, Mattila VM. Hospitalizations due to allergic reactions in Finnish and Swedish children during 1999-2011. *Allergy* (2016) **71**:677–83. doi:10.1111/all.12837

14. Lauritano EC, Novi A, Santoro MC, Casagranda I. Incidence, clinical features and management of acute allergic reactions: The experience of a single, Italian Emergency Department. *Eur Rev Med Pharmacol Sci* (2013) **17 Suppl 1**:39–44.

15. Lee S, Hess EP, Lohse C, Gilani W, Chamberlain AM, Campbell RL. Trends, characteristics, and incidence of anaphylaxis in 2001-2010: A population-based study. *J Allergy Clin Immunol* (2017) **139**:182-188.e2. doi:10.1016/j.jaci.2016.04.029

16. Lin RY, Cannon AG, Teitel AD. Pattern of hospitalizations for angioedema in New York between 1990 and 2003. *Ann Allergy Asthma Immunol* (2005) **95**:159–66. doi:10.1016/S1081-1206(10)61206-9

17. Lin RY, Shah SN. Increasing hospitalizations due to angioedema in the United States. *Ann Allergy Asthma Immunol* (2008) **101**:185–92. doi:10.1016/S1081-1206(10)60208-6

18. Lin RY, Anderson AS, Shah SN, Nurruzzaman F. Increasing anaphylaxis hospitalizations in the first 2 decades of life: New York State, 1990 -2006. *Ann Allergy Asthma Immunol* (2008) **101**:387–93. doi:10.1016/S1081-1206(10)60315-8

19. Ma L, Danoff TM, Borish L. Case fatality and population mortality associated with anaphylaxis in the United States. *J Allergy Clin Immunol* (2014) **133**:1075–83. doi:10.1016/j.jaci.2013.10.029

20. Manivannan V, Hyde RJ, Hankins DG, Bellolio MF, Fedko MG, Decker WW, et al. Epinephrine use and outcomes in anaphylaxis patients transported by emergency medical services. *Am J Emerg Med* (2014) **32**:1097–102. doi:10.1016/j.ajem.2014.05.014

21. McIntyre CL, Sheetz AH, Carroll CR, Young MC. Administration of epinephrine for life-threatening allergic reactions in school settings. *Pediatrics* (2005) **116**:1134–40. doi:10.1542/peds.2004-1475

22. Moro Moro M, Tejedor Alonso MA, Esteban Hernández J, Múgica García MV, Rosado Ingelmo A, Vila Albelda C. Incidence of anaphylaxis and subtypes of anaphylaxis in a general hospital emergency department. *J Investig Allergol Clin Immunol* (2011) **21**:142–9.

23. Motosue MS, Bellolio MF, van Houten HK, Shah ND, Campbell RL. Increasing Emergency Department Visits for Anaphylaxis, 2005-2014. *J Allergy Clin Immunol Pract* (2017) **5**:171-175.e3. doi:10.1016/j.jaip.2016.08.013

24. Mulla ZD, Simon MR. Hospitalizations for anaphylaxis in Florida: Epidemiologic analysis of a population-based dataset. *Int Arch Allergy Immunol* (2007) **144**:128–36. doi:10.1159/000103224

25. Mulla ZD, Simons FE. Concomitant chronic pulmonary diseases and their association with hospital outcomes in patients with anaphylaxis and other allergic conditions: A cohort study. *BMJ Open* (2013) **3**. doi:10.1136/bmjopen-2013-003197

26. Mullins RJ. Anaphylaxis: Risk factors for recurrence. *Clin Exp Allergy* (2003) **33**:1033–40.

27. Mullins RJ. Paediatric food allergy trends in a community-based specialist allergy practice, 1995-2006. *Med J Aust* (2007) **186**:618–21.

28. Mullins RJ, Clark S, Camargo CA. Regional variation in epinephrine autoinjector prescriptions in Australia: More evidence for the vitamin D-anaphylaxis hypothesis. *Ann Allergy Asthma Immunol* (2009) **103**:488–95. doi:10.1016/S1081-1206(10)60265-7

29. Mullins RJ, Dear KB, Tang ML. Time trends in Australian hospital anaphylaxis admissions in 1998-1999 to 2011-2012. *J Allergy Clin Immunol* (2015) **136**:367–75. doi:10.1016/j.jaci.2015.05.009

30. Mullins RJ, Wainstein BK, Barnes EH, Liew WK, Campbell DE. Increases in anaphylaxis fatalities in Australia from 1997 to 2013. *Clin Exp Allergy* (2016) **46**:1099–110. doi:10.1111/cea.12748

31. Norredam M, Sheikh A, Dynnes Svendsen K, Holm Petersen J, Garvey LH, Kristiansen M. Differences in hospital attendance for anaphylaxis between immigrants and non-immigrants: A cohort study. *Clin Exp Allergy* (2016) **46**:973–80. doi:10.1111/cea.12719

32. Poulos LM, Waters AM, Correll PK, Loblay RH, Marks GB. Trends in hospitalizations for anaphylaxis, angioedema, and urticaria in Australia, 1993-1994 to 2004-2005. *J Allergy Clin Immunol* (2007) **120**:878–84. doi:10.1016/j.jaci.2007.07.040

33. Pourang D, Batech M, Sheikh J, Samant S, Kaplan M. Anaphylaxis in a health maintenance organization: International Classification of Diseases coding and epinephrine auto-injector prescribing. *Ann Allergy Asthma Immunol* (2017) **118**:186-190.e1. doi:10.1016/j.anai.2016.10.027

34. Simons FE, Peterson S, Black CD. Epinephrine dispensing patterns for an out-of-hospital population: A novel approach to studying the epidemiology of anaphylaxis. *J Allergy Clin Immunol* (2002) **110**:647–51.

35. Smit DV, Cameron PA, Rainer TH. Anaphylaxis presentations to an emergency department in Hong Kong: Incidence and predictors of biphasic reactions. *J Emerg Med* (2005) **28**:381–8. doi:10.1016/j.jemermed.2004.11.028

36. Stewart AG, Ewan PW. The incidence, aetiology and management of anaphylaxis presenting to an accident and emergency department. *QJM* (1996) **89**:859–64.

37. Tanno LK, Molinari N, Bruel S, Bourrain JL, Calderon MA, Aubas P, et al. Field-testing the new anaphylaxis' classification for the WHO International Classification of Diseases-11 revision. *Allergy* (2017) **72**:820–6. doi:10.1111/all.13093

38. Tejedor Alonso MA, Moro Moro M, Hernández JE, Múgica García MV, Albelda CV, Ingelmo AR, et al. Incidence of anaphylaxis in hospitalized patients. *Int Arch Allergy Immunol* (2011) **156**:212–20. doi:10.1159/000322997

39. Tejedor Alonso MA, Moro Moro M, Múgica García MV, Esteban Hernández J, Rosado Ingelmo A, Vila Albelda C, et al. Incidence of anaphylaxis in the city of Alcorcon (Spain): A population-based study. *Clin Exp Allergy* (2012) **42**:578–89. doi:10.1111/j.1365-2222.2012.03930.x

40. Tejedor Alonso MA, Moro Moro M, Mosquera González M, Rodriguez-Alvarez M, Pérez Fernández E, Latasa Zamalloa P, et al. Increased incidence of admissions for anaphylaxis in Spain 1998-2011. *Allergy* (2015) **70**:880–3. doi:10.1111/all.12613

41. Tham EH, Tay SY, Lim DL, Shek LP, Goh AE, Giam YC, et al. Epinephrine auto-injector prescriptions as a reflection of the pattern of anaphylaxis in an Asian population. *Allergy Asthma Proc* (2008) **29**:211–5. doi:10.2500/aap.2008.29.3102

42. Turner PJ, Gowland MH, Sharma V, Ierodiakonou D, Harper N, Garcez T, et al. Increase in anaphylaxis-related hospitalizations but no increase in fatalities: An analysis of United Kingdom national anaphylaxis data, 1992-2012. *J Allergy Clin Immunol* (2015) **135**:956-63.e1. doi:10.1016/j.jaci.2014.10.021

43. van der Klauw MM, Stricker BH, Herings RM, Cost WS, Valkenburg HA, Wilson JH. A population based case-cohort study of drug-induced anaphylaxis. *Br J Clin Pharmacol* (1993) **35**:400–8.

44. Yang MS, Kim JY, Kim BK, Park HW, Cho SH, Min KU, et al. True rise in anaphylaxis incidence: Epidemiologic study based on a national health insurance database. *Medicine (Baltimore)* (2017) **96**:e5750. doi:10.1097/MD.0000000000005750

45. Yocum MW, Butterfield JH, Klein JS, Volcheck GW, Schroeder DR, Silverstein MD. Epidemiology of anaphylaxis in Olmsted County: A population-based study. *J Allergy Clin Immunol* (1999) **104**:452–6.

46. Yoon L, Kim BR, Lee JY, Kim K, Kim YM, Kim SH, et al. Clinical features of anaphylaxis according to age in a single university hospital in Korea. *Asian Pac J Allergy Immunol* (2017) **35**:96–101. doi:10.12932/AP0767

47. World Population Review. *Istanbul Population* (2017) [cited 2017 Dec 30]. Available from: http://worldpopulationreview.com/world-cities/istanbul-population/

48. United States Census Bureau. *1990 Census of Population: General Population Characteristics* [cited 2017 Dec 30]. Available from: https://census.gov/library/publications/1992/dec/cp-1.html

49. Mulla ZD, Lin RY, Simon MR. Perspectives on anaphylaxis epidemiology in the United States with new data and analyses. *Curr Allergy Asthma Rep* (2011) **11**:37–44. doi:10.1007/s11882-010-0154-7

50. United States Census Bureau. *New York - State Facts for Students* (2022) [cited 2022 Sep 05]. Available from: https://www.census.gov/schools/facts/new%20york

51. United States Census Bureau. *American FactFinder - Results* [cited 2017 Dec 30]. Available from: https://factfinder.census.gov/faces/tableservices/jsf/pages/productview.xhtml?pid=DEC_00_SLDH_DP1&prodType=table

52. Office for National Statistics. *Population Estimates for UK, England and Wales, Scotland and Northern Ireland* [cited 2017 Dec 30]. Available from: https://www.ons.gov.uk/peoplepopulationandcommunity/populationandmigration/populationestimates/datasets/populationestimatesforukenglandandwalesscotlandandnorthernireland

53. Worldometers. *Netherlands Population* [cited 2017 Dec 30]. Available from: http://www.worldometers.info/world-population/netherlands-population/

54. *Australian Historical Population Statistics, 2006* (2023) [cited 2023 Mar 17]. Available from: https://www.abs.gov.au/AUSSTATS/abs@.nsf/DetailsPage/3105.0.65.0012006?OpenDocument

55. Worldometers. *Australia Population* [cited 2017 Dec 30]. Available from: http://www.worldometers.info/world-population/australia-population/

56. Florida Department of Health. *FLHealthCHARTS - Population Query System* (2022) [cited 2022 Sep 05]. Available from: https://www.flhealthcharts.gov/FLQuery_New/Population/Count

57. Worldometers. *South Korea Population* [cited 2017 Dec 30]. Available from: http://www.worldometers.info/world-population/south-korea-population/

58. United States Census Bureau. *County Intercensal Tables: 2000-2010* [cited 2017 Dec 30]. Available from: https://www.census.gov/data/tables/time-series/demo/popest/intercensal-2000-2010-counties.html

59. United States Census Bureau. *County Intercensal Tables 1980-1990* [cited 2017 Dec 30]. Available from: https://www.census.gov/data/tables/time-series/demo/popest/1980s-county.html

60. *Scotland's Population 2005: The Registrar General's Annual Review of Demographic Trends: 151st Edition* (2014) [cited 2023 Mar 17]. Available from: https://www.nrscotland.gov.uk/files/statistics/scotlands-population-2005-the-register-generals-annual-review-151stedition/j9085e04.htm

61. Harduar-Morano L, Simon MR, Watkins S, Blackmore C. Algorithm for the diagnosis of anaphylaxis and its validation using population-based data on emergency department visits for anaphylaxis in Florida. *J Allergy Clin Immunol* (2010) **126**:98-104.e4. doi:10.1016/j.jaci.2010.04.017

62. Nicklas RA, Bernstein IL, Li JT. The diagnosis and management of anaphylaxis. Joint Task Force on Practice Parameters, American Academy of Allergy, Asthma and Immunology, American College of Allergy, Asthma and Immunology, and the Joint Council of Allergy, Asthma and Immunology. *J Allergy Clin Immunol* (1998) **101**:S465-528.

63. Sampson HA, Muñoz-Furlong A, Campbell RL, Adkinson NF, Bock SA, Branum A, et al. Second symposium on the definition and management of anaphylaxis: Summary report - Second National Institute of Allergy and Infectious Disease/Food Allergy and Anaphylaxis Network symposium. *J Allergy Clin Immunol* (2006) **117**:391–7. doi:10.1016/j.jaci.2005.12.1303

64. Simons FE, Ardusso LR, Bilò MB, El-Gamal YM, Ledford DK, Ring J, et al. World allergy organization guidelines for the assessment and management of anaphylaxis. *World Allergy Organ J* (2011) **4**:13–37. doi:10.1097/WOX.0b013e318211496c
